# Prognostic impact of *EGFR* mutation in non-small-cell lung cancer patients with family history of lung cancer

**DOI:** 10.1371/journal.pone.0177015

**Published:** 2017-05-09

**Authors:** Jung Soo Kim, Min Seong Cho, Jong Hyeon Nam, Hyun-Jung Kim, Kyeng-Won Choi, Jeong-Seon Ryu

**Affiliations:** Dept. of Internal Medicine, Inha University Hospital, 27, Inhang-Ro, Jung Gu, Incheon, South Korea; Queen Mary Hospital, HONG KONG

## Abstract

**Background:**

A family history can be a valuable tool in the era of precision medicine. Although a few studies have described an association of family history of lung cancer with *EGFR* activating mutation, their impact on survival of lung cancer patients is unclear.

**Methods:**

The study included consecutive 829 non-small-cell lung cancer patients who received analysis of *EGFR* mutation in a prospective lung cancer cohort. Family history of lung cancer was obtained by face-to-face interviews at the time of diagnosis. An association of *EGFR* activating mutation with a family history of lung cancer in first-degree relatives was evaluated with multivariate logistic regression analysis, and its association with survival was estimated with Cox’s proportional hazards model.

**Results:**

Seventy five (9.0%) patients had family history of lung cancer. The *EGFR* mutation was commonly observed in patients with positive family history compared to those with no family history (46.7% *v* 31.3%, χ^2^ p = 0.007). The family history was significantly associated with the *EGFR* mutation (aOR and 95% CI: 2.01 and 1.18–3.60, p = 0.011). Patients with the positive family history survived longer compared to those without (MST, 17.9 *v* 13.0 months, log-rank p = 0.037). The presence of the *EGFR* mutation was associated with better survival in patients without the family history (aHR and 95% CI: 0.72 and 0.57–0.90, p = 0.005). However, this prognostic impact was not observed in patients with the positive family history (aHR and 95% CI: 1.01 and 0.50–2.36, p = 0.832).

**Conclusions:**

In comparison to patients without the family history, *EGFR* activating mutation was common, and it did not affect prognosis in patients with positive family history.

## Introduction

Lung cancer is the most common cause of cancer-related death worldwide [1]. Non-small-cell lung cancer (NSCLC) accounts for 80–85% of lung cancers. Histology-based cytotoxic chemotherapy and molecular targeted therapy are a mainstay in treating NSCLC patients, recently incorporating immunotherapy as well [2]. In spite of these advances, the prognosis for patients remains grave.

In the era of precision medicine, the importance of accurate characterization of clinical phenotype as well as genetic diversity is increasing. In this regard, family histories of lung cancer have been thought to add information in predicting risk for lung cancer and prevalence of mutations on epidermal growth factor receptor (EGFR) gene. Having a family history of lung cancer from first-degree relatives confers increased risk for lung cancer [3–6]. Activating mutations on *EGFR* gene are more commonly observed in the patients with the family history compared to those without the history [7–9]. The T790M or V843I mutation on *EGFR* gene was reported to be inherited, so the family history may have an effect on the susceptibility of *EGFR* tyrosine kinase inhibitors (TKIs) [10, 11]. However, the effect of the family history on patient survival has been investigated in a few studies where the association was inconsistent [12, 13]. Moreover, the prognostic impact of *EGFR* mutations by the family history has not been examined.

Therefore, we conducted a study to investigate the association of family history of lung cancer with *EGFR* activating mutation and the prognostic impact in a prospective lung cancer cohort.

## Patients and methods

### Study population

This study initially considered 902 consecutive NSCLC patients who were histologically diagnosed in Inha University Hospital from January 2006 through January 2014 and received *EGFR* mutational testing. To maintain quality of information, patients whose family histories were not obtained (n = 12), who failed to test *EGFR* mutational status due to poor quality of DNA (n = 11), or who were transferred to other hospitals just after diagnosis (n = 50) were all excluded. Finally, 829 patients were included in this study. All information about the clinical characteristics of the patients was obtained from the Inha Lung Cancer Cohort, where clinical information was prospectively collected [14]. This study was approved by the institutional review board of Inha University hospital, and informed consent by patients was waived.

### *EGFR* mutation analysis

Mutational testing was performed by ISU ABXIS Co. Ltd (Seoul, South Korea), an independent commercial laboratory before November 2011, and then by the Department of Pathology at the Inha University Hospital. Mutational analysis was performed to detect mutations in exons 18–21 of the *EGFR* gene by directional sequencing of polymerase chain reaction fragments amplified with DNA from paraffin-embedded tissue obtained at the time of the diagnosis.

### Clinical variables and survival measurement

Information on age, sex, smoking habits, Eastern Cooperative Oncology Group (EGOG) performance status, body mass index, and histology were obtained at the time of the diagnosis. A family history of lung cancer was obtained by face-to-face interviews by a research nurse (HJK). Presence of the family history was defined when any first-degree relatives of the patients had a history of lung cancer. First-degree relatives included a parent, sibling or offspring of the patients. The stages of all patients were estimated according to the 7th edition of TNM classification [15]. Treatments given to the patients were classified into treatment and no treatment. Palliative treatment including radiation therapy to metastatic site or supportive care was classified as no treatment.

Overall survival was measured as an outcome and estimated from the time of diagnosis until death as a result of all causes. In all, 541 patients died. The date of death was obtained by medical records of our hospital or by contacting relatives of the patients. We could not obtain survival of six patients who were lost at follow-up after hospital discharge.

### Statistical methods

Distributions of clinical variables between patients by family history were compared using χ^2^ tests. The association of family history with *EGFR* activating mutation was evaluated through multivariate logistic regression testing after adjusting by other clinical variables. The association of family history on overall survival was estimated using the Kaplan-Meier method and log-rank testing. Hazard ratios (HRs) and 95% confidence intervals (CIs) were calculated using the Cox`s proportional hazard model. Null hypotheses of no difference were rejected if p-values were less than .05, or, equivalently, if the 95% CIs of risk point estimates excluded 1. All analyses were performed using IBM’s SPSS statistical software package (SPSS Inc. Released 2009. PASW Statistics for Windows, Version 18.0. Chicago: SPSS Inc.).

## Results

### Patient characteristics

Table 1 displays the clinical characteristics of a total of 829 NSCLC patients by presence of family history of lung cancer in first-degree relatives. Seventy five (9%) patients had a positive family history. The median age of the patients was 66.5 years. There were 535 men (64.5%), 553 ever-smokers (66.9%) and 537 adenocarcinomas (64.8%). The *EGFR* mutation was found in 271 (37.2%) patients: 132 (48.7%) patients carried the L858R mutation on exon 21; 112 carried the exon 19 deletion (41.3%); and others numbered 27 (10.0%). Of the patients with the *EGFR* mutation, 162 (59.8%) patients received EGFR-TKIs treatment where 113 (20.2%) patients not having the mutation received the EGFR-TKIs treatment (data not shown). Use of EGFR-TKIs was not different between patients with or without family history of lung cancer (χ^2^ p = 0.446).

**Table 1:** Characteristics of 829 non-small-cell lung cancer patients by family history of lung cancer.

		Family history of lung cancer		
Variables	Total	Presence	Absence	p
Age at diagnosis, median	66.5	66.6	66.0	0.636
Sex				
Male	535 (64.5)	49 (65.3)	486 (64.5)	0.880
Female	294 (35.5)	26 (34.7)	268 (35.5)	
Smoking habit				
Ever	553 (66.9)	48 (64.0)	505 (67.2)	0.569
Never	273 (33.1)	27 (36.0)	246 (32.8)	
ECOG performance status				
0–1	582 (70.7)	54 (72.0)	528 (70.6)	0.798
2–4	241 (29.3)	21 (28.0)	220 (29.4)	
Body mass index (m^2^/Kg)				
< 22.4	336 (41.6)	29 (39.2)	307 (41.8)	0.661
≥ 22.4	472 (58.4)	45 (60.8)	427 (58.2)	
Histology				
Adenocarcinoma	537 (64.8)	55 (73.3)	482 (63.9)	0.104
Non-adenocarcinoma	292 (35.2)	20 (26.7)	272 (36.1)	
EGFR activating mutations				
Positive	271 (37.2)	35 (46.7)	236 (31.3)	0.007
Negative	558 (67.3)	40 (53.3)	518 (68.7)	
Stage				
I-II	241 (29.1)	24 (32.4)	217 (28.8)	0.509
III-IV	587 (70.9)	50 (67.6)	537 (71.2)	
EGFR tyrosine kinase inhibitors				
Yes	275 (33.4)	28 (37.3)	247 (33.0)	0.446
No	549 (66.6)	47 (62.7)	502 (67.0)	
Treatment				
Yes	659 (79.9)	63 (84.0)	596 (79.5)	0.350
No	166 (20.1)	12 (16.0)	154 (20.5)	

ECOG = Eastern Cooperative Oncology Group; Body mass index by 22.4 of median value

### Association of family history of lung cancer and *EGFR* activating mutation

Distribution of the L858R mutation and exon 19 deletion did not differ between patients by family history of lung cancer (χ^2^ p = 0.448). The *EGFR* mutation was commonly observed in patients with family history of lung cancer compared to those not having the family history (χ^2^ p = 0.007). After adjusting for age, sex, smoking habits, the ECOG performance status, histology, and stages, we found that positive family history was significantly associated with presence of *EGFR* activing mutation (aOR and 95% CI: 2.01 and 1.18–3.60, p = 0.011) (Table 2).

**Table 2:** Clinical variables for predicting *EGFR* activating mutations: Multiple logistic regression analysis results.

Variables	ORs	95% CIs	P
Family history of lung cancer (yes *v* no)	2.01	1.18–3.60	0.011
Age (increasing)	1.00	0.98–1.01	0.947
Gender (female *v* male)	2.07	1.20–3.61	0.011
Smoking habit (never *v* ever smoker)	2.64	1.50–4.67	0.001
ECOG performance status (0–1 *v* 2–4)	0.97	0.65–1.46	0.893
Body mass index (< 22.4 *v* ≥ 22.4)	1.03	0.72–1.47	0.872
Histology (adenocarcinoma *v* non-adenocarcinoma)	3.60	2.31–5.60	< 0.001
Stages (I and II *v* III and IV)	1.23	0.84–1.81	0.291

ECOG = Eastern Cooperative Oncology Group; Body mass index by 22.4 of median value

### Impact of *EGFR* activating mutation on overall survival of patients by family history of lung cancer

**Fig 1:**
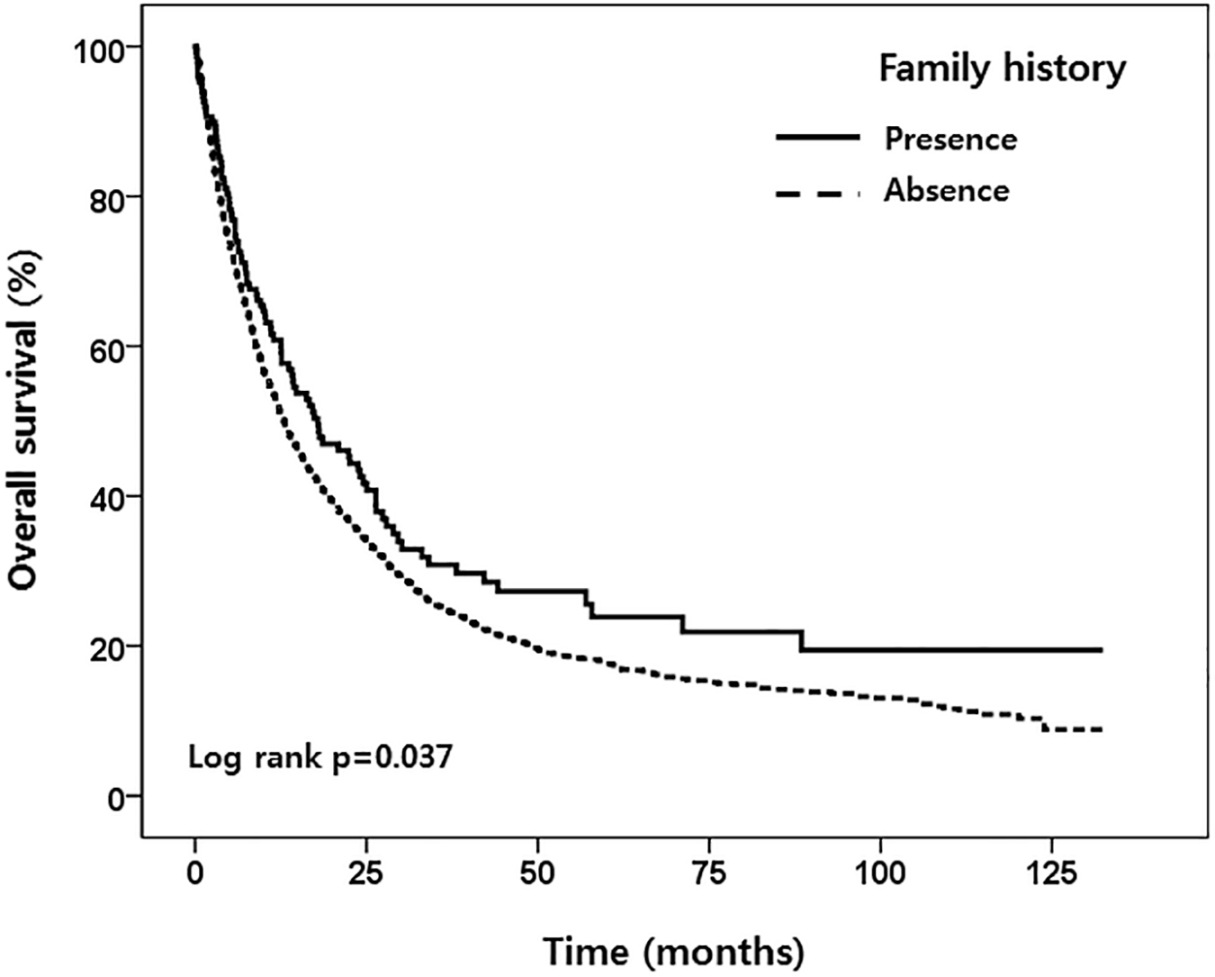
Impact of family history of lung cancer on survival of patients with non-small-cell lung cancer.

Patients with family history of lung cancer survived longer compared to those without the family history (MST, 17.9 *v* 13.0 months, log-rank p = 0.037) (Fig 1).

However, this impact disappeared when adjusting for age, sex, smoking habits, the ECOG performance status, histology, stages, and treatment (aHR and 95% CI, 0,97 and 0.70–1.34, p = 0.840). As expected, patients with *EGFR* activating mutation survived significantly longer compared to those having the wild type *EGFR* gene (MST, 13.3 *v* 30.9 months, log-rank p < 0.001) (data not shown). When the patients were classified into two groups by family history, *EGFR* activating mutation was significantly associated with survival in the patients with no family history (aHR and 95% CI: 0.72 and 0.57–0.90, p = 0.005), while this was not observed in patients with positive family history (aHR and 95% CI: 1.01 and 0.50–2.36, p = 0.832) (Table 3).

**Table 3:** Impact of *EGFR* activating mutation on survival of non-small-cell lung cancer patients by family history of lung cancer: Multivariate analysis results.

Family history of lung cancer	aHRs (95% CIs) *	p
Presence		
EGFR mutation Negative	1.00	0.832
Positive	1.01 (0.50–2.36)	
Absence		
EGFR mutation Negative	1.00	0.005
Positive	0.72 (0.52–0.90)	

*adjusted for age, sex, smoking habit, ECOG performance status, body mass index, histology, and stage.

## Discussion

For the first time, we demonstrate that *EGFR* mutations are prognostic in NSCLC patients without family history of lung cancer in first-degree relatives but not in patients with a positive family history in this study.

In a few studies on prevalence of *EGFR* mutations or prognostic impact of family history, information on family history was obtained from reviewing medical records retrospectively, and the prevalence varied from 5.1 to 26.4 percent [7, 9, 12, 13, 16] (Table 4).

**Table 4:** Summary of studies of the association between family history and *EGFR* activating mutations or survival in lung cancer patients.

	Population (n)	%, having family history (relatives defined)	*EGFR* activating mutation (%, in patients having *v* not having family history)	Effect of family history on survival (aHR and 95% CI)
**This study**	NSCLC (n = 829)	9.0 (first-degree)	Association with higher prevalence (46.7 *v* 31.3,)	No association with overall survival (0.97 and 0.70–1.34)
**He et al. [7]** **(2013)**	NSCLC (n = 538)	7.9 (first-degree)	Association with higher prevalence (49.6 *v* 38.5)	N/E
**Cheng et al. [9] (2015)**	NSCLC, never smokers (n = 246)	15.8 (first- and second-degree)	Association with higher prevalence (71.7 *v* 25.4)	N/E
**Li et al. [12]** **(2011)**	NSCLC (n = 4,491)	5.1 (first- and second-degree)	N/E	Association with better overall survival (0.69 and 0.51–0.93)
**Ganti et al. [13] (2009)**	NSCLC and SCLC (n = 560)	26.4 (first-, second-, and third-degree)	N/E	Association with a poorer overall survival (1.65 and 1.07–2.56)

N/E, not evaluated

Family history was prospectively ascertained through face-to-face interviews with patients or their family by a well-trained registered nurse in this study. Family history of lung cancer was asked only in first-degree relatives because recall bias can make family history of second-degree or more distant relatives inaccurate. This study shows that the prevalence of family history of lung cancer in first-degree relatives is 9%. In addition, it is indicative of the proportion of *EGFR* activating mutations in the patients with the family history.

Two studies have reported the proportion of *EGFR* activating mutations by family history [7,9]. This study confirms that family history of lung cancer in first-degree relatives is significantly associated with the presence of *EGFR* activating mutation. Why patients with family history of lung cancer have a higher prevalence of *EGFR* activating mutation has been poorly understood. However, accumulating genetic evidence can explain familial aggregation in a variety of cancers, including lung cancer. In NSCLC patients with family history of lung cancer, familial aggregations were noted in genetic variants located in the chromosomal region 15q24-25.1 [17].

As for the effect of family history of lung cancer on survival of the patients, it has been examined in two different cohorts where the prognostic impact was contrasted, and *EGFR* activating mutations were not considered [12, 13]. This study showed an intermediate result in that patients with a positive family history survive longer compared to patients with no family history in univariate analysis. However, the prognostic impact disappeared after adjustments with variables. Nevertheless, it is the strength of this study that the clinical information including family history is carefully scrutinized to make sure this association.

*EGFR* activating mutations are associated with prognosis in patients with lung cancer [18]. However, its impact is not observed in patients with the family history when patients are divided by the family history in this study. Germ-line mutations of *EGFR* gene such as T790M or V843I can begin to explain familial aggregation of lung cancer patients; its presence is important in developing resistance to EGFR-TKIs [10, 11, 19–22]. Prevalence of germ-line T790M mutation in patients with *EGFR* activating mutation varies with sensitivity of detection methods so the range is 0.4–3% using Sanger sequencing but it increases up to 34.2% with more sensitive methods such as next-generation sequencing (NGS) [23]. Although it is not clear whether using high-sensitive techniques to detect low copy number of T790M mutations is related to clinical outcome in NSCLC patients, *EGFR* activating mutation without T790M mutation is associated with longer survival than those with T790M mutation [23]. Given that *EGFR* activating mutations were more likely in patients having the germ-line *EGFR* mutations-related to poor prognosis [10, 11, 21], this coincidence could provide a plausible explanation on the prognostic impact of *EGFR* activation mutations by the family history in this study.

There are some limitations to this study. First, direct squencing was used to detect mutations on *EGFR* gene in this study. The patients were diagnosed from January 2006 through January 2014, when direct sequencing was the standard method. It is a less sensitive method in detecting the mutations than NGS analysis so that prevalence of the germ-line mutation is too low to analyze their effect. Therefore, it is a challenge to demonstrate familial aggregation of the germ-line mutation or their association with survival by the interaction of family history and *EGFR* activating mutations. Nevertheless, the extrapolation from results of past studies may offer a rational explanation [19, 23, 24]. Second, overall survival was estimated, but progression-free survival was not in this retrospective study. Overall survival is a gold standard for prognostic impact. Progression-free survival is the best option for evaluating the effect of EGFR-TKIs. However, the reliability of the results is always a problem in a retrospective study.

In conclusion, this is the first study to demonstrate that the presence of *EGFR* activating mutation is associated with better overall survival in NSCLC patients without family history of lung cancer. However, the prognostic impact disappears in patients with a positive family history. Thus, family history needs to be considered when treating with EGFR-TKIs.
